# Inversion of Circularly Polarized Luminescence in the Left‐Handed Chitosan‐Templated Co‐assemblies

**DOI:** 10.1002/advs.202415260

**Published:** 2025-01-31

**Authors:** Yu An, Zhaocun Shen, Fang Zhang, Qiuya Yang, Zihan Han, Mingjie Wang, Hongze Ma, Linjie Yu, Wei Yuan, Kunyan Sui

**Affiliations:** ^1^ State Key Laboratory of Bio‐Fibers and Eco‐textiles College of Materials Science and Engineering Key Laboratory of Shandong Provincial Universities for Advanced Fibers and Composites Qingdao University 308 Ningxia Road Qingdao 266071 P. R. China; ^2^ School of Chemistry Chemical Engineering and Biotechnology Nanyang Technological University 21 Nanyang Link Singapore 637371 Singapore

**Keywords:** chiral assembly, chitosan, circularly polarized luminescence, J‐aggregates

## Abstract

Circularly polarized luminescence (CPL) materials are attractive due to their unique applications in fields such as 3D displays, information encryption, and chiroptical switches. Natural biomolecules‐based CPL materials are gaining plenty of attention due to their chiral diversity and sustainability. However, it is still challenging to construct CPL materials with opposite CPL signs from a single natural biomolecule due to its inherent chirality. Here, chiral assemblies with opposite CPL signs using chitosan oligosaccharide (COS) and achiral luminescent dyes are successfully prepared. It is found that COS can serve as a chiral template to induce the ordered assembly of the dyes along the polymer chain through electrostatic attraction interaction. It is demonstrated experimentally that the structural planarity of the dye molecules is crucial for the formation of chiral co‐assemblies. Interestingly, the left‐handed COS‐templated co‐assemblies can emit CPL with opposite handedness, which is controlled by the helicity degree of the co‐assemblies. This study not only deepens the understanding of the complex assembly of natural biomacromolecules but also provides new insights into the design and construction of CPL materials.

## Graphical abstract

Left‐handed helical J‐aggregates with circularly polarized luminescence (CPL) activity are successfully constructed by the templated assembly of achiral anionic luminescent dye (CBS) on the cationic chitosan oligosaccharide (COS) via electrostatic attraction interaction. Interestingly, the CPL inversion of the helical J‐aggregates is realized by regulating the stoichiometric ratio of COS/CBS and the aging time of the assembly.

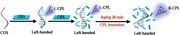

## 1 Introduction

Circularly polarized luminescence (CPL) refers to the phenomenon that chiral compounds emit left and right‐handed circularly polarized light of different intensities under unpolarized light excitation.^[^ ^1^, ^2^, ^3^, ^4^, ^5^ ^]^ CPL‐active materials are attracting more and more attention due to their promising applications in 3D display, information encryption, chiroptical switches, and smart materials.^[^ ^5^, ^6^, ^7^, ^8^, ^9^, ^10^, ^11^, ^12^, ^13^, ^14^, ^15^ ^]^ CPL‐active materials are categorized into organic molecules,^[^ ^16^, ^17^, ^18^, ^19^ ^]^ lanthanide complexes,^[^ ^20^, ^21^, ^22^, ^23^, ^24^, ^25^ ^]^ inorganic nanomaterials,^[^ ^26^, ^27^, ^28^, ^29^, ^30^ ^]^ and assembled systems.^[^ ^31^, ^32^, ^33^, ^34^, ^35^, ^36^, ^37^, ^38^, ^39^, ^40^, ^41^, ^42^ ^]^ Among these CPL‐active materials, assembled systems can integrate molecular diversity and functionality through supramolecular strategies, thus achieving effective regulation and amplification of CPL performance.^[^ ^33^, ^34^, ^42^ ^]^ In general, these assembled CPL systems are constructed by the self‐assembly of chiral building blocks or the co‐assembly of chiral and achiral building blocks via non‐covalent interactions.^[^ ^1^, ^43^, ^44^ ^]^ Especially, the chiral co‐assembly strategy offers an abundance of opportunities for the rational design and preparation of CPL‐active materials from various achiral luminescent substances.^[^ ^4^ ^]^ Typically, natural biomacromolecules, such as DNA and polysaccharides, can serve as excellent chiral templates to co‐assemble with achiral luminophores to construct chiral materials with CPL activity.^[^ ^45^, ^46^, ^47^, ^48^, ^49^, ^50^ ^]^ This method avoids the tedious chemical synthesis process of chiral building blocks. For example, Duan's group made use of DNA to co‐assemble with achiral cyanine molecules through electrostatic interactions, thereby inducing strong CPL.^[^ ^48^ ^]^ Deng's group co‐assembled cellulose nanocrystals with achiral fluorescent polymer to prepare a CPL composite film.^[^ ^51^ ^]^ However, owing to the inherent single helical sense of natural biomacromolecules, they typically emit CPL with single‐handedness, which is not suitable for practical applications. In this regard, it is still challenging to prepare CPL‐active materials with opposite CPL properties from a single natural biomacromolecule.

**Scheme 1:**
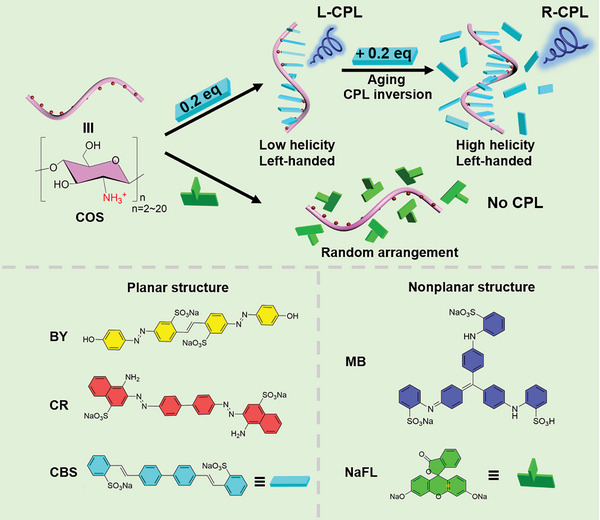
Schematic illustration of COS‐templated assembly with dye molecules and the CPL inversion. The planar molecule CBS can assemble along the helical COS template to form left‐handed J‐aggregates, which emit opposite CPL signals depending on the equivalents of CBS and aging time.

Polysaccharides are a kind of important biomacromolecule, possessing the advantages of biocompatibility, biodegradability, and sustainability.^[^ ^52^ ^]^ Chitosan is the only positively charged polysaccharide in nature. It is helical conformationally and has abundant chiral sites and ammonium groups, making it an excellent candidate as a template for chiral co‐assembly. Herein, we report the inversion of CPL in the left‐handed chitosan‐templated co‐assemblies (**Scheme** **1**). Chitosan oligosaccharide (COS) is used as a chiral template to induce the ordered assembly of achiral dyes along the polymer chain through electrostatic attraction interaction. It is found that the structural planarity of the dye molecules affects their stacking along the COS chains. Interestingly, the COS‐templated co‐assemblies can emit CPL with opposite signs, which is dependent on the equivalents of fluorescent dyes and the aging time. This study demonstrates the feasibility that opposite CPL activity can be achieved from a single natural macromolecule.

## 2 Results and Discussion

### 2.1 Co‐assembly and Chiral Transfer of COS with Dyes

**Figure 1:**
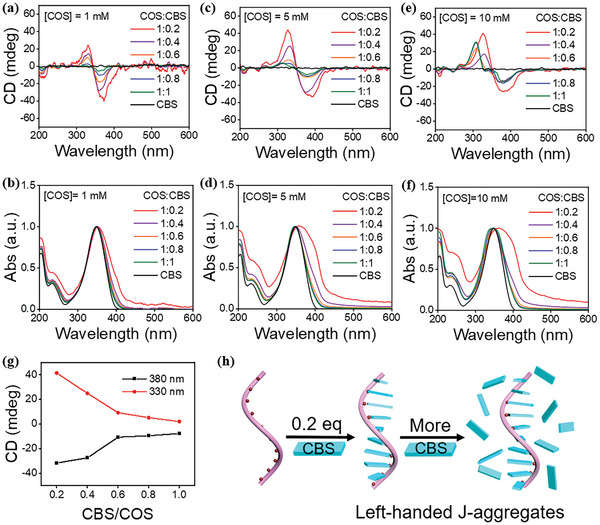
Normalized CD and UV–vis spectra of CBS solution and COS/CBS mixtures upon mixing at different COS_unit_/CBS molar ratios with different COS concentrations: a,b) [COS]_unit_ = 1 mm. c,d) [COS]_unit_ = 5 mm. e,f) [COS]_unit_ = 10 mm. g) Plots of CD intensity at 380 nm and 330 nm of COS/CBS suspensions as a function of CBS/COS_unit_ molar ratios. [COS]_unit_ = 5 mm. h) Schematic representation of the COS‐templated co‐assembly with CBS into a left‐handed helix upon mixing.

In order to obtain chiral co‐assemblies from chitosan and oppositely charged dye molecules, a series of dropwise addition mixing experiments were conducted to identify the optimal assembly conditions. To ensure the solubility of chitosan in water, COS with a molecular weight of less than or equal to 2K and a concentration of no >10 mm (0.1, 1, 5, 10 mm) was selected for the co‐assembly with the fluorescent dye disodium 4,4'‐bis(2‐sulfonatostyryl)biphenyl (CBS). COS and CBS aqueous solutions were prepared by heating under stirring. Subsequently, CBS solution was dropwise added into COS solutions according to different COS_unit_/CBS molar ratios (1:0.2, 1:0.4, 1:0.6, 1:0.8, 1:1; COS_unit_ represents the monosaccharide repeating unit). Notably, it was observed that the COS/CBS mixtures transitioned from a clear solution to an opalescent suspension during the dropwise addition process (Figure S1, Supporting Information). The ground state chirality of the COS/CBS suspensions was characterized by circular dichroism (CD) spectroscopy. The spectral results showed that pure COS solution exhibited no CD signal and absorption band in the UV–vis spectral range. CBS solution displayed an intense absorption band at 347 nm but did not demonstrate inherent CD activity due to the lack of a chiral center (Figure S2, Supporting Information). However, obvious CD signals were detected at the CBS absorption wavelength of 347 nm from the COS/CBS suspensions with different COS concentrations (except 0.1 mm) and different COS_unit_/CBS molar ratios (**Figure** **1**a,c,e), indicating the formation of chiral COS/CBS co‐assemblies. The absence of a CD signal at 0.1 mm COS is probably due to the concentration being too low to assemble with the dye molecules. (Figure S3, Supporting Information). Moreover, the comparative analysis indicated that COS could transfer its chirality to CBS more effectively at a COS concentration of 5 mm (Figure S4, Supporting Information).

The CD and UV–vis spectra of the COS/CBS suspensions at a COS concentration of 5 mm were carefully analyzed in order to reveal their assembly mechanism. COS/CBS suspensions displayed strong negative and positive Cotton bands at 380 and 330 nm, respectively, with a zero crossing at 347 nm (Figure 1c), suggesting that CBS molecules were arranged in a left‐handed helical pattern in the COS/CBS co‐assemblies.^[^ ^53^ ^]^ Meanwhile, the UV–vis absorption spectrum of the COS/CBS suspension at a COS_unit_/CBS molar ratio of 1:0.2 exhibited a red shift and a broadened absorption band compared to that of the CBS solution (Figure 1d), indicating that the formation of J‐type aggregate of CBS on the COS template.^[^ ^54^, ^55^, ^56^, ^57^, ^58^, ^59^ ^]^ Note that the CD intensity was strongest and the red‐shift of the absorption band was largest at a COS_unit_/CBS molar ratio of 1:0.2 for all the suspensions (Figure 1a–g). The CD intensity and the turbidity of the suspensions were decreased with increasing the equivalents of CBS (Figures 1g and S5, Supporting Information), indicating that excess CBS molecules existed in the monomeric state and did not contribute to the real CD intensity. Scanning electron microscopy (SEM) characterization indicated that the morphology of the COS/CBS co‐assemblies is the cluster‐like aggregate (Figure S6, Supporting Information), suggesting that COS/CBS chains intertwined with each other. According to the above results, we proposed the co‐assembly and chirality transfer mechanism as follows: when a small amount of CBS is added to the COS solution, COS functions as a helical template to induce the helical stacking of CBS molecules along the COS chain in a left‐handed helix sense via electrostatic attraction interaction (Figure 1h). However, if excess CBS molecule is added, it will exist in the monomeric state, causing the absorption spectra of the samples to get close to that of CBS monomer.

In order to deeply explore the mechanism of COS‐templated chiral co‐assembly, we further performed contrast experiments using other dyes, including congo red (CR), brilliant yellow (BY), fluorescein sodium salt (NaFL) and methyl blue (MB) (Figure S7, Supporting Information). Interestingly, CD signals were observed from the CD spectra of both COS/CR and COS/BY suspensions (Figure S8, Supporting Information), while COS/NaFL solution and COS/MB suspension did not show any CD signal (Figure S9, Supporting Information). Moreover, the COS/CR and COS/BY system also showed molar ratios‐dependent CD spectral change, which was similar to the COS/CBS system. Further to note, CBS, CR, and BY are planar molecules, whereas NaFL and MB have a nonplanar molecular structure. In principle, compared with planar dyes (CBS, CR, and BY), nonplanar dyes (NaFL and MB) should own larger steric hindrance, which is unfavorable for their ordered stacking. Thus, we speculated that the planar structure of the dye molecules is crucial for its chiral assembly on the COS template. For planar dyes such as CBS, they are assembled in an ordered helical stacking mode along the COS chains through electrostatic attraction interaction, thus making it easier to induce a chiral CD signal. However, nonplanar dyes such as NaFL, cannot form helically stacked assemblies with COS as proved by the absence of a CD signal (Figure S9, Supporting Information), which is probably due to their larger steric hindrance.

### 2.2 Influence Factors of COS‐Templated Chiral Co‐assembly

In order to investigate the effect of COS molecular weights on the COS‐templated chiral co‐assembly, various molecular weights of COS (3K to 4K, 5K to 6K, and 7K), all of which are completely soluble in water, were used to mix with CBS. CD spectra showed that COS with different molecular weights always displayed CD signals after mixing with CBS (Figure S10, Supporting Information). We found that the CD intensity was also strongest at a COS_unit_/CBS molar ratio of 1:0.2 for COS with different molecular weights, which was consistent with that of COS with a 2K molecular weight. Additionally, COS with a 2K molecular weight had a significantly higher CD signal than that of COS with higher molecular weights (Figure S11, Supporting Information). This result suggested that COS with lower molecular weights can form more ordered chiral assemblies with CBS. As the molecular weight increases, the increase in the length of the molecular chains may lead to the entanglement of the chains, which may interfere with the effective chiral co‐assembly of COS with CBS, leading to a weaker CD signal.

**Figure 2:**
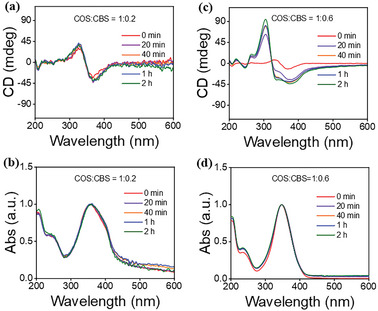
CD and UV–vis spectra of COS/CBS suspensions at COS_unit_/CBS molar ratios of a,b) 1:0.2 and (c,d) 1:0.6 at different aging times. [COS]_unit_ = 5 mm.

We also found that the CD signals of chiral suspensions are related to the aging time. For the COS/CBS suspension with the strongest CD signal at a COS_unit_/CBS molar ratio of 1:0.2, its turbidity, CD intensity, and UV–vis absorption band remained essentially unchanged with increasing aging time (**Figures** **2**a,b and S12a, Supporting Information). However, for the samples with more CBS, for example COS_unit_/CBS molar ratio of 1:0.6, the turbidity of the suspension decreased with increasing aging time (Figure S12b, Supporting Information). Interestingly, the CD signal was enhanced over time, and the zero‐crossing point of the CD signal shifted from 347 nm to 325 nm, accompanied by a slight broadening of the UV–vis absorption spectra (Figure 2c,d). We calculated the molar extinction coefficients at 347 nm of CBS and COS/CBS assemblies at a molar ratio of 1:0.2 according to the data in Figure 1d and found that the molar extinction coefficient of CBS (6.04 × 10^5^ M^−1^ cm^−1^) is approximately three times larger than that of COS/CBS assemblies (2.19 × 10^5^ M^−1^ cm^−1^). At the molar ratio of 1:0.6, excess CBS existed in its monomeric state in the system. Therefore, we think that the absorption band of the COS/CBS assemblies is likely to be masked by the excess CBS monomer with a larger molar extinction coefficient. As a result, only a slight broadening of the UV–vis absorption spectra is observed with aging, as shown in Figure 2d. These results suggested that the helical stacking pattern of CBS molecules along the COS chains changes to some extent with aging although the change in UV–vis spectra is very small. Thus, the CD signal was not only affected by the initial COS_unit_/CBS molar ratio but also was correlated with the aging time.

### 2.3 CPL Emission of COS‐Templated Co‐Assemblies and its Inversion

**Figure 3:**
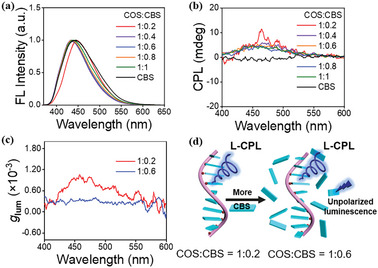
a) Normalized fluorescence spectra of COS/CBS suspensions at different COS_unit/_CBS molar ratios. λ_ex_ = 347 nm, [COS]_unit_ = 5 mm. b) Normalized CPL spectra of COS/CBS suspensions upon mixing at different COS_unit_/CBS molar ratios. λ_ex_ = 347 nm. c) The *g*_lum_ values of COS/CBS suspensions upon mixing at COS_unit_/CBS molar ratios of 1:0.2 and 1:0.6. [COS]_unit_ = 5 mm. d) Schematic representation of the CPL emission with the same handedness but different *g*_lum_ values from the left‐handed helical co‐assemblies at COS_unit_/CBS molar ratios of 1:0.2 and 1:0.6 without aging.

We characterized the chiroptical characteristics of COS‐templated assemblies using CPL spectroscopy. Under 365 nm UV light irradiation, COS/CBS suspension emitted a blue fluorescence (Figure S13, Supporting Information). The COS/CBS suspensions showed fluorescence emission at 450 nm when excited at a wavelength of 347 nm (**Figure** **3**a). Meanwhile, confocal fluorescence microscopy images also confirmed that the COS/CBS suspensions emitted blue fluorescence (Figure S14, Supporting Information), which was consistent with the result from fluorescence spectra. The CPL spectra of CBS or COS solution did not show any CPL signal (Figures 3b and S15, Supporting Information). In contrast, COS/CBS suspensions upon mixing displayed positive CPL signals around 450 nm, and the strongest CPL signal was found at a COS_unit_/CBS molar ratio of 1:0.2 (Figure 3b). This is consistent with the corresponding CD result discussed previously, where the CD signal is also strongest at the molar ratio of 1:0.2 without aging (Figure 1c). The luminescence dissymmetry factor (*g*_lum_) is used to assess the quality of CPL, which can be estimated using the equation: *g*_lum_ = 2 × (*I*_L –_ *I*_R_) /(*I*_L_ + *I*_R_), where *I*_L_ and *I*_R_ correspond to the intensities of left and right‐handed circularly polarized light, respectively. The calculated *g*_lum_ values of the COS/CBS suspensions at COS_unit_/CBS molar ratios of 1:0.2 and 1:0.6 at 450 nm were +0.94 × 10^−3^ and +0.31 × 10^−3^, respectively (Figure 3c). Figure 3d presents a schematic representation of left‐handed CPL emission from the left‐handed helical co‐assemblies without aging. When the molar ratio of COS_unit_/CBS is increased from 1:0.2 to 1:0.6, CBS molecules unabsorbed onto the COS chain emit non‐polarized light, causing the decrease of *g*_lum_ values. The chiroptical properties of the COS/NaFL mixtures were also characterized and the results showed that no CPL signal was generated (Figure S16, Supporting Information), which is consistent with the absence of CD signals in the COS/NaFL mixtures discussed previously (Figure S9, Supporting Information).

**Figure 4:**
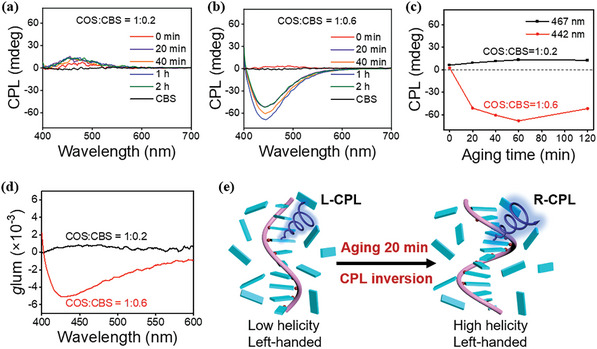
a,b) Normalized CPL spectra of COS/CBS suspensions at COS_unit/_CBS molar ratios of a) 1:0.2 and b) 1:0.6 at different aging times. [COS]_unit_ = 5 mm, λ_ex_ = 347 nm. c) Plots of CPL intensity for COS/CBS suspensions at COS_unit_/CBS molar ratios of 1:0.2 (467 nm) and 1:0.6 (442 nm) as a function of aging time. d) The *g*_lum_ values of COS/CBS suspensions at COS_unit_/CBS molar ratios of 1:0.2 and 1:0.6 aged for 1 h. e) Schematic representation of the CPL signal inversion induced by the helicity increasing of COS‐templated co‐assembly with the aging time. The COS_unit_/CBS molar ratio is 1:0.6.

Considering that the CD signs of COS/CBS co‐assemblies were related to the aging time, we also measured their CPL spectra at different aging times. At a COS_unit_/CBS molar ratio of 1:0.2, the suspension always showed positive CPL signals as the aging time was prolonged (**Figure** **4**a), which is consistent with its unchanged CD signals (Figure 2a). Unexpectedly, when more CBS was added at the COS_unit_/CBS molar ratio of 1:0.6, the initial positive weak CPL signal reversed to a negative strong CPL signal after aging 20 minutes (Figure 4b,c), corresponding to the aforementioned shifting and enhancing of the CD signals after aging (Figure 2c). In addition, the suspension samples with molar ratios higher than 1:0.2 (1:0.4, 1:0.8, and 1:1) also showed a reversal of the CPL signal with aging time (Figure S17, Supporting Information). As shown in Figure 4d, the *g*_lum_ values of the suspensions at COS_unit_/CBS molar ratios of 1:0.2 and 1:0.6 aged for 1 hour were +0.94 × 10^−3^ and −4.25 × 10^−3^, respectively. Based on the above results, we proposed a mechanism for the CD enhancement and CPL inversion (Figure 4e). At higher molar ratios (> 1:0.2), the excess anionic CBS molecules around the left‐handed COS/CBS co‐assemblies could screen the positive charges of the COS chain and thus reduce the electrostatic repulsion along the COS polymer backbone, causing the COS chain to contract to give higher helicity over time. As a result, the CD intensity of COS/CBS co‐assemblies is enhanced with aging. The CPL inversion and enhancement of COS/CBS co‐assemblies is also caused by its helicity increasing with aging although the helical sense of the co‐assemblies remains left‐handed. Therefore, the CPL signs of this kind of biomacromolecule‐based co‐assemblies can be easily regulated and reversed by the molar ratios of the biomacromolecule to a fluorescent dye and the aging time even though the biomacromolecule has inherent chirality.

## 3 Conclusion

In conclusion, biomacromolecule‐templated assemblies with opposite CPL properties were successfully constructed by controlling the molar ratios and the aging time. The experimental results revealed that fluorescent dyes could assemble along the helical COS chains to form left‐handed J‐aggregates through electrostatic attraction interaction. It was further found that the planar structure of dye molecules plays a key role during the chiral assembly and transfer process. Most importantly, the reversal of CPL signals in the COS‐templated chiral assemblies could be achieved by prolonging the aging time when dye molecules are in excess. This study provides new insights into the regulation of biomacromolecule‐based CPL materials.

## Conflict of Interest

The authors declare no conflict of interest.

## Supporting information



Supporting Information

## Data Availability

The data that support the findings of this study are available in the supplementary material of this article.
