# Supplementary material for: Inversion of Circularly Polarized Luminescence in the Left‐Handed Chitosan‐Templated Co‐assemblies
Source: Adv Sci (Weinh). 2025 Jan 31;12(12):2415260. doi: 10.1002/advs.202415260 (PMC11948070; doi:10.1002/advs.202415260)
Supplement: Supplementary file 1 — Supporting Information [file ADVS-12-2415260-s001.docx]

Supporting Information

**Inversion of Circularly Polarized Luminescence in the Left-Handed Chitosan-Templated Co-assemblies**

*Yu An, Zhaocun Shen^*^, Fang Zhang, Qiuya Yang, Zihan Han, Mingjie Wang, Hongze Ma, Linjie Yu, Wei Yuan, and Kunyan Sui^*^*

**Table of Contents**

1. Experimental section 2

1.1 Materials 2

1.2 Instruments 2

- 1. Methods 2

1. Supplementary Figures 3
2. **Experimental section**

**1.1 Materials**

All reagents and solvents were commercially available and used without further purification, unless otherwise noted. Chitosan oligosaccharide (COS) and congo red (CR) were purchased from Macklin Reagent Co., Ltd. 4,4'-bis(2-sulfostyrylene-based) biphenyl disodium salt (CBS), bright yellow (BY), fluorescein sodium salt (NaFL) and methyl blue (MB) were purchased from Aladdin Chemistry Co., Ltd. Milli-Q water (18.2 MΩ·cm) was used in all cases.

**1.2 Instruments**

Circular dichroism (CD) and UV-vis spectra were acquired using JASCO J-1500 spectropolarimeters. Circular polarized luminescence (CPL) spectra were acquired using JASCO CPL-300 instrument. The fluorescence spectra of the suspensions were measured on an F-7100 fluorescence spectrophotometer using a Xenon lamp as the excitation light source. Luminescence images were taken with an Axio-Imager-LSM-800 confocal microscope. SEM images were obtained on a JSM-7800F scanning electron microscope. Before SEM measurements, the samples on silicon wafers were coated with a thin layer of Pt to increase the contrast.

**1.3 Methods**

**Preparation of COS aqueous solutions.** COS powder (16.0 mg) was dissolved in water (10 mL) to form a COS aqueous solution (10 mM) under stirring. COS aqueous solutions with lower concentrations were obtained by diluting the 10 mM COS aqueous solution.

**Preparation of dye aqueous solutions .** CBS powder (45.0 mg ) was dissolved in water (4 mL) to form a CBS aqueous solution (20 mM). CR , BY, NaFL and MB aqueous solutions (20 mM) were prepared in the same way.

**Preparation of COS/dye mixtures.** Typically, a certain volume of CBS solutions was dropwise added into different concentrations of COS aqueous solutions at different COS_unit_/CBS molar ratios (1:0.2, 1:0.4, 1:0.6, 1:0.8, and 1:1), yielding COS/CBS solutions or suspensions.

1. **Supplementary Figures**


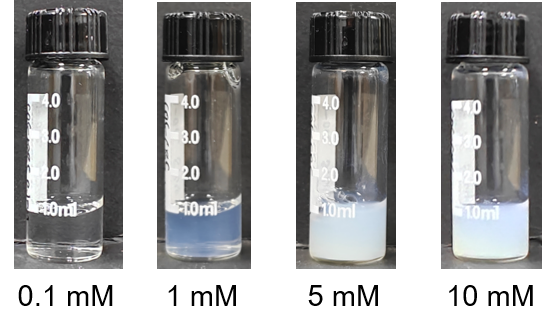


**Figure S1.** Photographs of the COS/CBS mixtures at different COS concentrations. [COS]_unit_ = 0.1 mM, 1 mM, 5 mM and 10 mM. COS_unit_/CBS = 1:0.2.


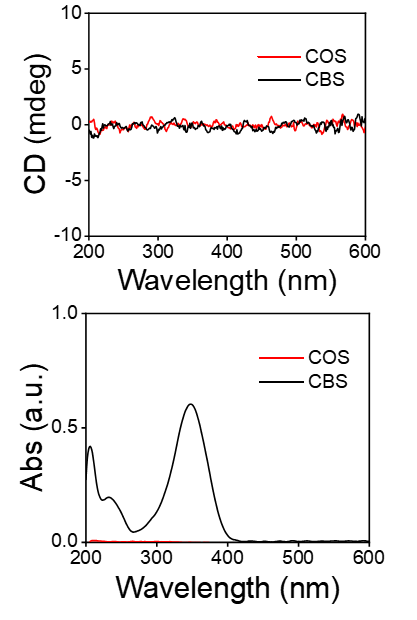


Figure S2. CD and UV-vis spectra of COS solution and CBS solution. [COS]_unit_ = 5 mM. [CBS] = 1mM.


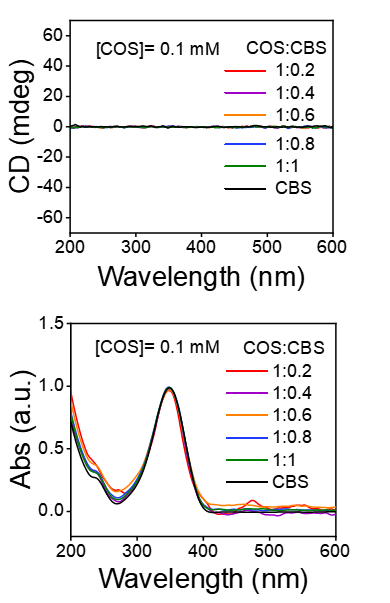


**Figure S3.** Normalized CD and UV-vis spectra of CBS solution and COS/CBS mixtures at different COS_unit_/CBS molar ratios. [COS]_unit_ = 0.1 mM.

**
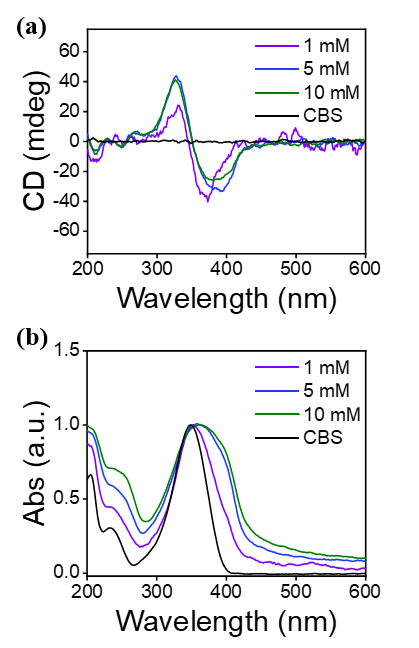
**

**Figure S4.** Normalized CD spectra (a) and UV-vis spectra (b) of COS/CBS suspensions at a COS_unit_/CBS molar ratio of 1:0.2 with different COS concentrations (1, 5, 10 mM).


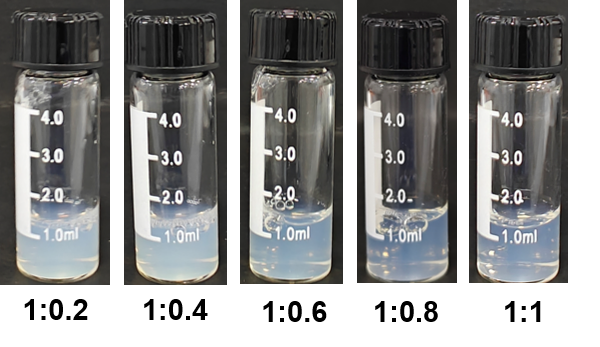


**Figure S5.** Photographs of COS/CBS suspensions at various COS_unit_/CBS molar ratios (1:0.2, 1:0.4, 1:0.6, 1:0.8, 1:1). [COS]_unit_ = 5 mM.


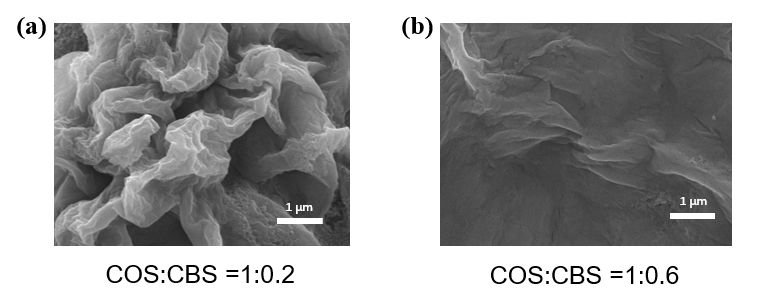


**Figure S6.** SEM images of the dried samples of COS/CBS suspensions at different COS_unit_/CBS molar ratios: (a) COS_unit_/CBS = 1:0.2; (b) COS_unit_/CBS = 1:0.6. [COS]_unit_ = 5 mM.


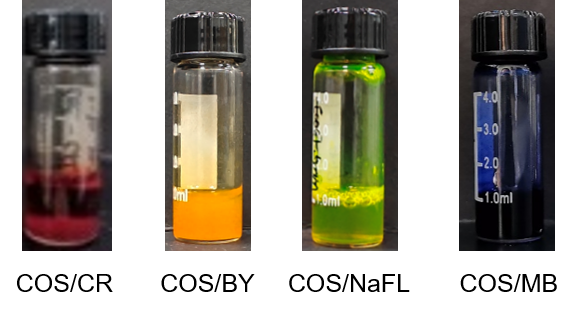


**Figure S7.** Photographs of the COS/dyes mixture with different dyes: CR, BY, NaFL and MB. COS_unit_/dye = 1:0.2. [COS]_unit_ = 5 mM.


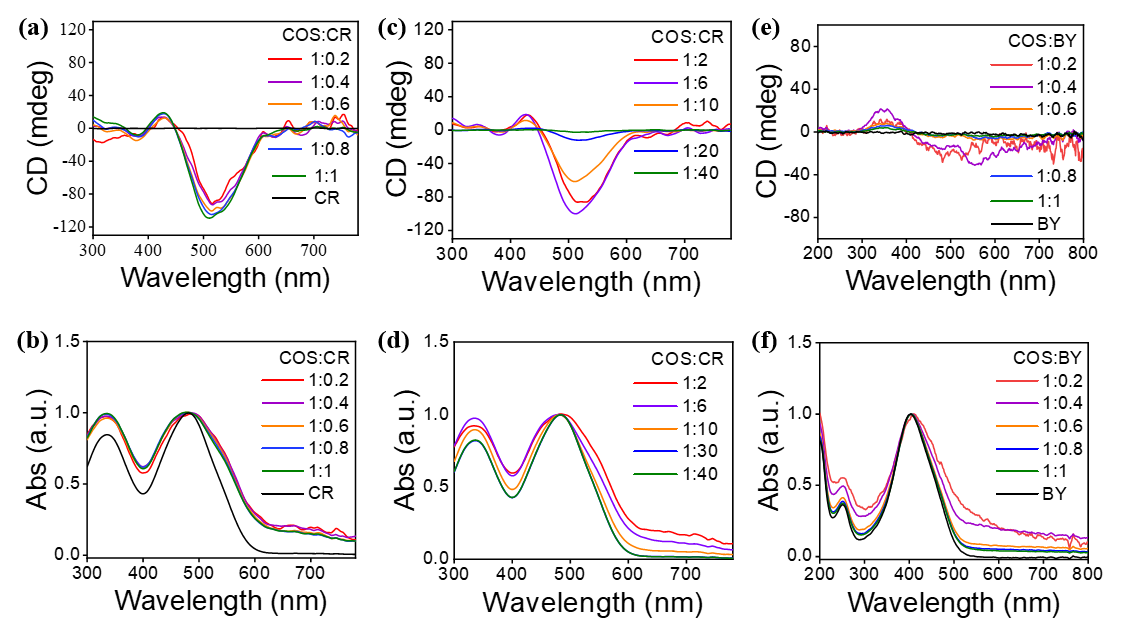


**Figure S8.** Normalized CD and UV-vis spectra of dye solutions and COS/dye suspensions at different COS_unit_/dye molar ratios with different dyes: (a-d) COS_unit_/CR, (e, f) COS_unit_/BY. [COS]_unit_ = 5 mM.


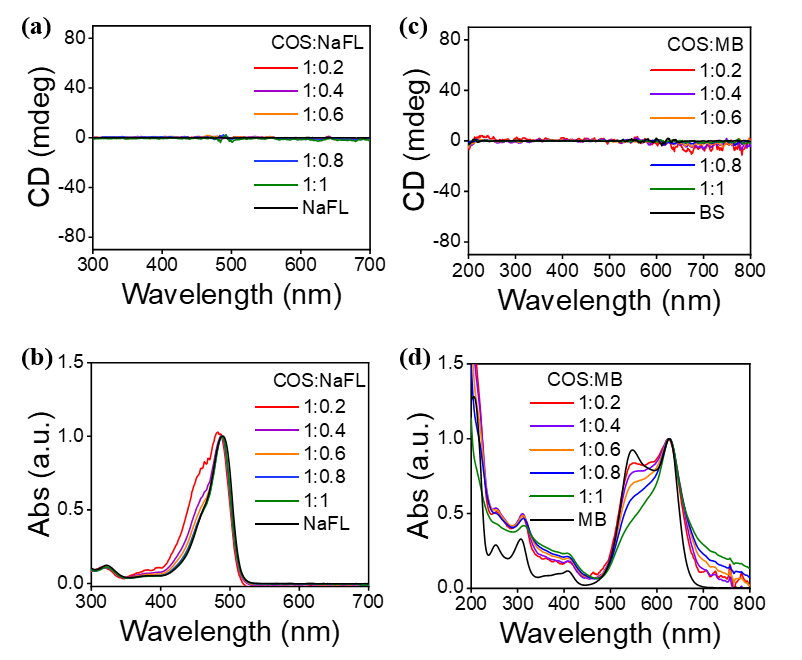


**Figure S9.** Normalized CD and UV-vis spectra of dye solutions and COS/dye suspensions at different COS_unit_/dye molar ratios with different dyes: (a, b) COS_unit_/NaFL, (c, d) COS_unit_/MB. [COS]_unit_ = 5 mM.

**
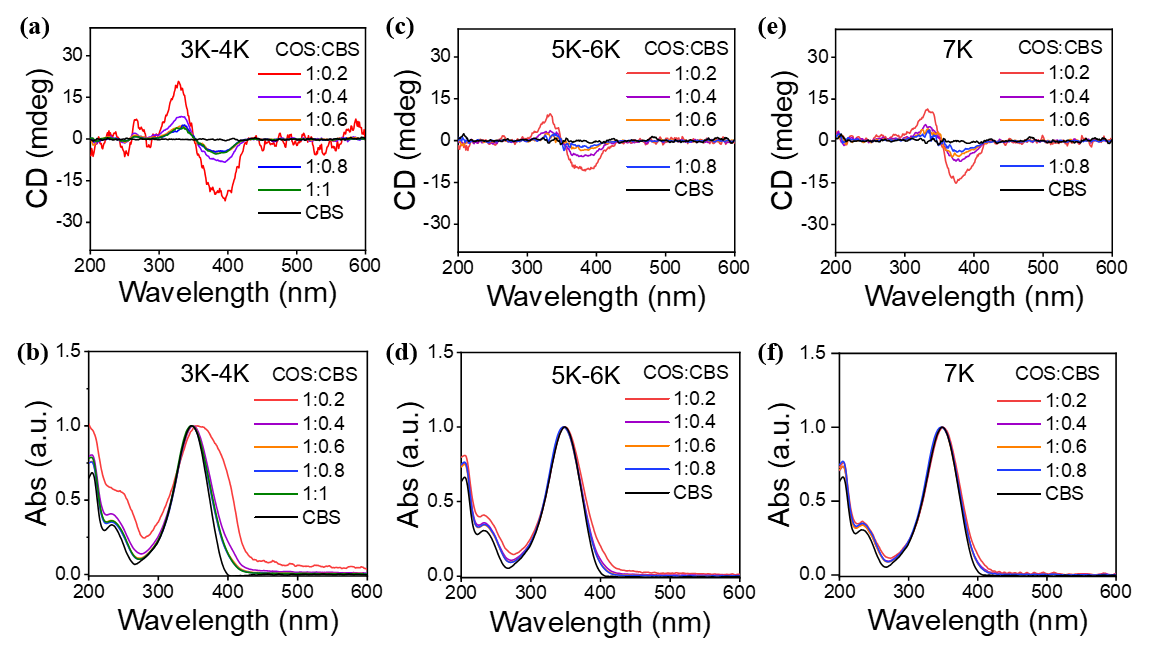
**

**Figure S10.** Normalized CD and UV-vis spectra of CBS solution and COS/CBS suspensions with different COS molecular weights: (a, b) 3K-4K, (c, d) 5K-6K and (e, f) 7K. [COS]_unit_ = 5 mM.


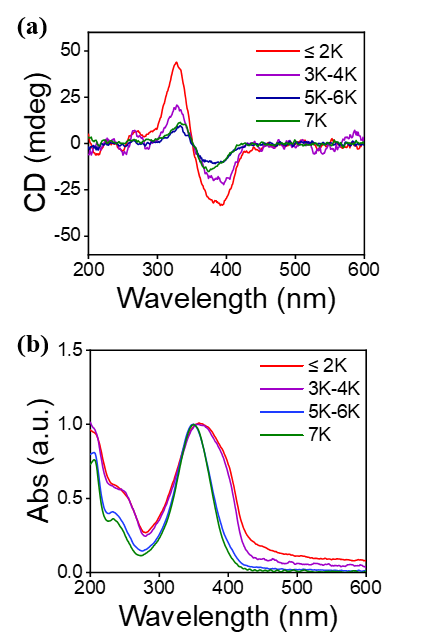


**Figure S11.** Normalized CD spectra (a) and UV-vis spectra (b) of COS/CBS suspensions at a COS_unit_/CBS molar ratio of 1:0.2 with different COS molecular weights: ≤2K, 3K-4K, 5K-6K, 7K. [COS]_unit_ = 5 mM.


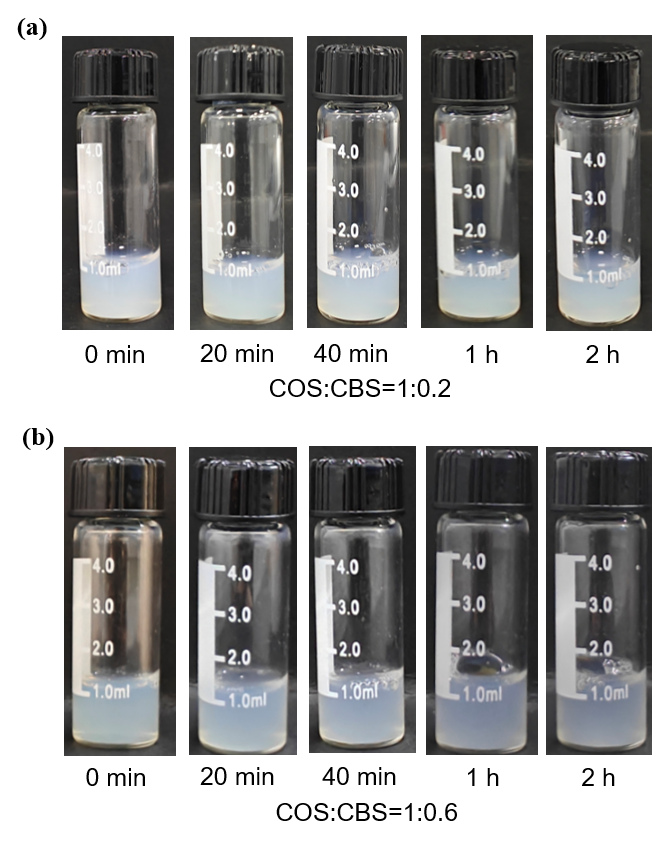


**Figure S12.** Photographs of the COS/CBS suspensions at different aging time: (a) COS_unit_/CBS = 1:0.2; (b) COS_unit_/CBS = 1:0.6. [COS]_unit_ = 5 mM.

**
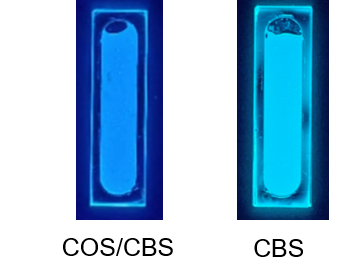
**

**Figure S13.** Photographs of COS/CBS suspension at a COS_unit_/CBS molar ratio of 1:0.2 and a CBS solution ([CBS] = 20 mM) under 365 nm UV light. [COS]_unit_ = 5 mM.


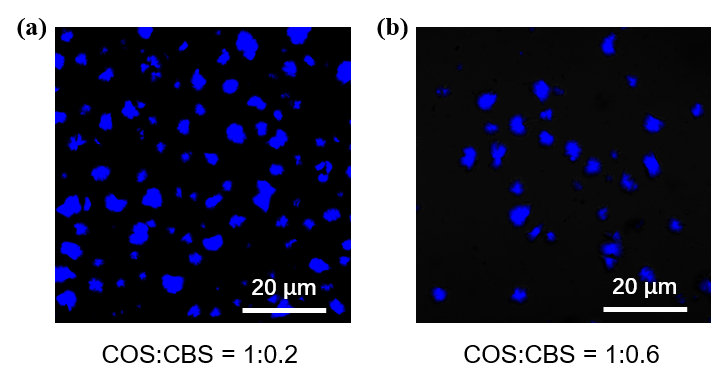


**Figure S14.** Confocal fluorescence microscopy images of the COS/CBS suspensions at COS_unit_/CBS molar ratios of (a) 1:0.2 and (b) 1:0.6. λ_ex_ = 401 nm.


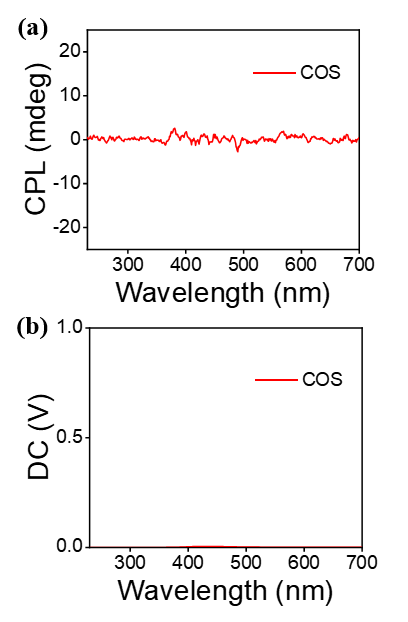


**Figure S15.** CPL spectra of COS solution. [COS]_unit_ = 5 mM, λ_ex_ = 210 nm.


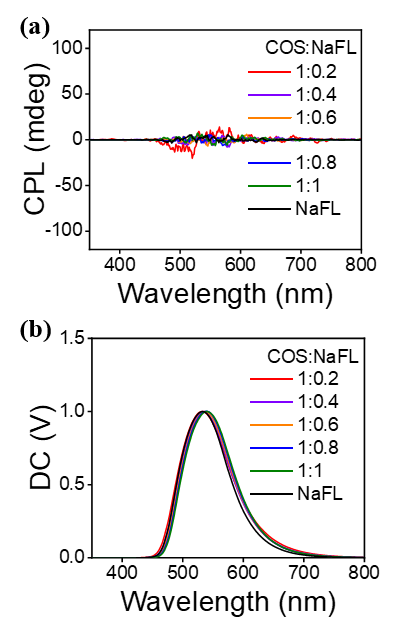


**Figure S16.** Normalized CPL spectra of COS/NaFL mixtures at different COS_unit_/NaFL molar ratios. λ_ex_ = 489 nm. [COS]_unit_ = 5 mM.


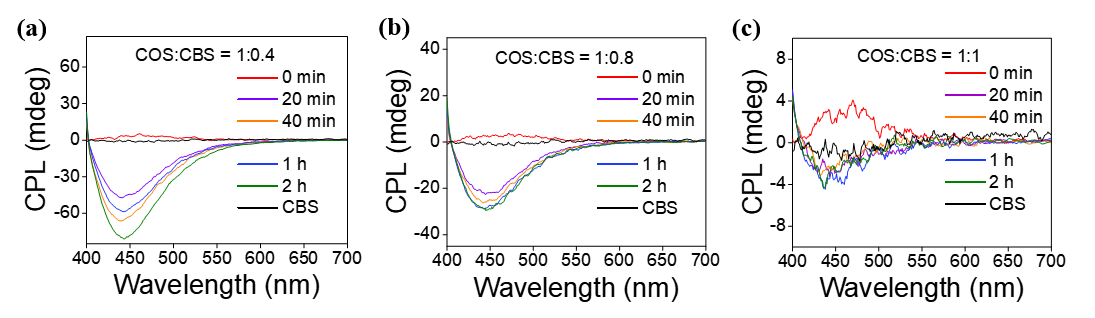


**Figure S17.** Normalized CPL spectra of COS/CBS suspensions at COS_unit_/CBS molar ratios of (a) 1:0.4, (b) 1:0.8 and (c) 1:1 at different aging time. [COS]_unit_ = 5 mM, λ_ex_ = 347 nm.
